# Introspective False Negative Prediction for Black-Box Object Detectors in Autonomous Driving

**DOI:** 10.3390/s21082819

**Published:** 2021-04-16

**Authors:** Qinghua Yang, Hui Chen, Zhe Chen, Junzhe Su

**Affiliations:** School of Automotive Studies, Tongji University, Shanghai 201804, China; 1510799@tongji.edu.cn (Q.Y.); 1651876@tongji.edu.cn (Z.C.); 1831611@tongji.edu.cn (J.S.)

**Keywords:** false negative prediction, introspection, failure prediction, object detection, autonomous driving

## Abstract

Object detection plays a critical role in autonomous driving, but current state-of-the-art object detectors will inevitably fail in many driving scenes, which is unacceptable for safety-critical automated vehicles. Given the complexity of the real traffic scenarios, it is impractical to guarantee zero detection failure; thus, online failure prediction is of crucial importance to mitigate the risk of traffic accidents. Of all the failure cases, False Negative (FN) objects are most likely to cause catastrophic consequences, but little attention has been paid to the online FN prediction. In this paper, we propose a general introspection framework that can make online prediction of FN objects for black-box object detectors. In contrast to existing methods which rely on empirical assumptions or handcrafted features, we facilitate the FN feature extraction by an introspective FN predictor we designed in this framework. For this purpose, we extend the original concept of introspection to object-wise FN predictions, and propose a multi-branch cooperation mechanism to address the distinct foreground-background imbalance problem of FN objects. The effectiveness of the proposed framework is verified through extensive experiments and analysis, and the results show that our method successfully predicts the FN objects with 81.95% precision for 88.10% recall on the challenging KITTI Benchmark, and effectively improves object detection performance by taking FN predictions into consideration.

## 1. Introduction

Object detection serves as a fundamental task in environment perception for autonomous driving, but state-of-the-art object detectors will inevitably fail in many scenarios [1,2,3,4], especially when the driving scenes are very different from those in the training dataset [5,6]. Typical failures are False Negatives (FNs) and False Positives (FPs), which refer to missed and false-alarmed objects respectively. These failures bring serious safety concerns to autonomous driving, and have already caused several catastrophic consequences [7]. While improving the performance of object detectors is necessary, it is incapable of ensuring the elimination of failures, especially under perceptually degraded circumstances. Thus, it is of vital importance to perform online failure prediction for autonomous driving, so the system can take appropriate operations as early as possible when failures are inevitable to happen.

To mitigate this problem, some prior works [1,8,9,10] are devoted to outputting the uncertainty information of the detection results during the online inference process, but these methods can only provide uncertainties for detected objects in the outputs, and cannot deal with FNs. Some studies [4,11] try to predict online performance metrics, such as Average Precision (AP), of an object detector; however, these predicted metrics can only reflect the overall detection performance on the input image, and cannot provide specific object-wise failure predictions, which we believe are more indispensable and constructive for the application of autonomous driving. Considering the grave consequences of FNs, some recent research [2,12,13] propose methods for online prediction of FNs, but these methods rely heavily on empirical assumptions or handcrafted FN features, which brings discernable limitations to their performance.

For the same concern of consequences caused by missed objects in autonomous driving, we prioritize FNs among other types of failures, and propose a general framework for online FN prediction in this paper.

**Figure 1:**
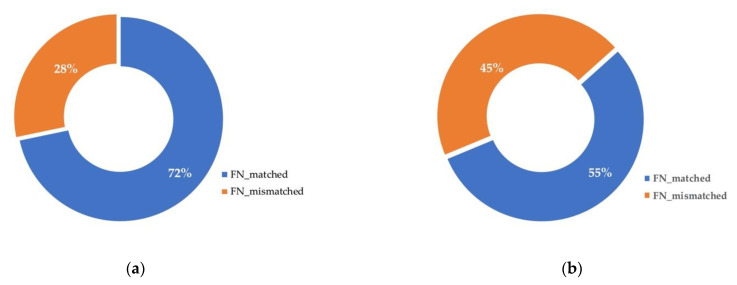
Relationship between FN statistics and common features considered to be FN-related. (**a**) FN statistics of RRC detector [2] on KITTI Tracking Dataset [14]; (**b**) FN statistics of SSD detector [15] on KITTI 2D Object Dataset [14]. Factors considered here include truncation (≥0.3), occlusion (≥2, largely occluded), depth (≥50 m), and height (≤25 px), the striped partitions show that for both detectors, more than 28% of FNs do not match any of these factors.

In contrast to prior insights, we do not think FNs can be properly described by handcrafted features. We notice that although there is correlation between FNs and factors such as occlusion, truncation, and scales [3], features based on these factors cannot provide proper FN descriptions. This is illustrated in Figure 1, we choose several factors which are commonly believed to be FN-related, and for two different object detectors, we count the proportions of their FNs that match none of these factors. The statistics show that more than 28% of FNs do not match any of these factors. This indicates that these factors are not sufficient and necessary conditions for FNs to happen. As it is impossible to exhaust all factors that might be the cause of FNs, it can be inferred that handcrafted features based on enumerated factors cannot effectively describe FNs, and will limit the performance when they are used for FN prediction.

**Figure 2:**
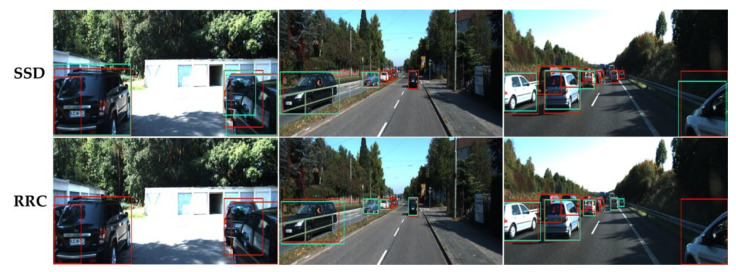
Visual comparisons of ground truth objects (red) and detected objects (green) produced by different object detectors. The first row shows ground truth objects and detections of SSD detector [15] on samples in KITTI Benchmark [14], and the second row shows ground truth objects and detections of RRC detector [2] on the same samples. It can be observed that FN objects missed in the same samples are different for the two detectors.

We also notice that FN objects in an image tend to be different for different detectors, as shown in Figure 2. This indicates that the detection characteristics of a given object detector should be taken into consideration when predicting FNs it missed, and that FN prediction methods should have generalization facility to different object detectors. To the best of our knowledge, no online FN prediction method has been introduced so far to provide object-wise FN predictions based on non-handcrafted features, and to be applicable to various object detectors as well.

**Figure 3:**
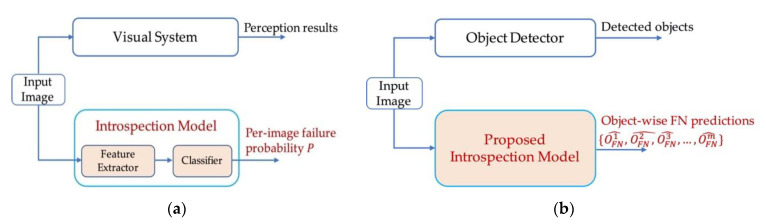
Original concept of introspection and our extension. (**a**) Original concept of introspection. Failure features are extracted and classified to obtain a per-image failure probability P; (**b**) Our extension of introspection. The concept of introspection is extended to perform object-wise failure predictions in our work, $\hat{O_{FN}^{m}}$ refers to the mth predicted FN object in the input image.

In this paper, we propose a general introspection framework to address the online prediction of FNs for black-box object detectors. The idea of introspection was first proposed by [16] referring to the self-assessment ability of a robot, and was used by [17] for online failure prediction of a visual system. As depicted in Figure 3a, this is done by learning a convolutional neural network (CNN) to extract failure features from a test image, and predict a failure probability for it. In doing so, this model, known as the introspection model, obtains self-evaluating facility for the given visual system. Based on this idea, we formulated the online FN predictor in the proposed framework as an introspection model, and train it to predict FNs based on the detection characteristics and FN features it learns of the given object detector. The advantages of doing so mainly lie in three aspects. Firstly, we do not have to “define” FN features by ourselves, or making assumptions on what FN objects should share in common, instead, we let the introspection model to “learn” to identify FN features for a given object detector, which makes our method not subject to the limitations caused by handcrafted features or empirical assumptions in previous methods. Secondly, we do not need to use additional algorithms to make FN proposals, because the introspection model can make direct predictions based on what it learns. Thirdly, since no underlying detail of the detector is used, our method has the generalization facility to black-box detectors.

Although the above introspection scheme in Figure 3a is feasible to predict failures online for black-box visual systems, we cannot directly apply it in the application of online FN prediction. This is because the original concept of introspection can only predict per-image failure probabilities. As depicted in Figure 3a, original introspection model is not able to account for the existence or locations of FN objects, while autonomous driving systems need object-wise FN predictions to make comprehensive decisions. Besides, FNs usually suffer from distinct foreground-background imbalance problem [18] in the image space, which makes object-wise FN predictions a challenging task.

To address the difficulties mentioned above, we propose two key designs for our introspection framework. Firstly, inspired by the anchor-free idea in [19,20], we extend the original concept of introspection to object-wise FN prediction, as the skeleton depicted in Figure 3b. Secondly, in order to cope with the foreground-background imbalance problem of FNs, we propose a multi-branch cooperation mechanism based on an auxiliary segmentation task, which reduces the searching space for finding FNs, and encourages the learning of consistent features in the training process. To verify the effectiveness of the proposed framework, we conduct extensive experiments and analysis for its FN prediction ability, as well as the improvements it brings to the object detector task.

To summarize, our main contributions are the following:

- We propose a general framework to achieve online prediction of FNs for black-box object detectors, which provides a new perspective where online FN prediction can be formulated as an introspection model.
- We extend the original concept of introspection to object-wise FN prediction, and propose a multi-branch cooperation mechanism to address the distinct foreground-background imbalance problem of FN objects.
- We verify the effectiveness of the proposed method through extensive experiments and analysis, and prove that considering FN predictions can effectively improve the safety of autonomous driving.

## 2. Related Works

### 2.1. Introspection

The concept of introspection in robotics is first introduced in [16] as a self-assessment mechanism for a robot to assist its decision-making. In later years, this concept has been interpreted and applied for failure prediction in different applications [16,17,21,22,23]. For example, Daftry et al. [17] described the robot introspection as the self-evaluating facility for a robot system to know when it does not know. To obtain self-evaluating ability for a vision-based autonomous navigation system, they trained a convolutional neural network (CNN) to extract features from image frames, and used these features as the input to a linear Support Vector Machine (SVM) classifier to generate a failure probability for a test image. High failure probability indicates unreliable predictions the visual system will make on the corresponding test image.

Kuhn et al. [24] applied the idea of introspection to predict future disengagements of a black-box autonomous driving system. They construct their introspection model as a Long Short-Term Memory (LSTM) classifier that can learn from the previous disengagement sequences of the system, and predict system failures several seconds in advance.

The studies mentioned above share two points in common. Firstly, they rely on raw image input for failure prediction, and do not need the underlying details of the vision system. Secondly, instead of providing more details about the failures, they can only predict a failure probability for a whole image input of the vision system, which is not sufficient for autonomous driving application that need more specific and detailed failure clues.

Kuhn et al. [25] extended the original concept of introspection to pixel-wise failure prediction for semantic image segmentation. They use the errors made by the given segmentation model, and train a decoder to predict pixel-wise segmentation errors. Our work also makes extensions for the original introspection, but instead of predicting pixel-wise segmentation errors, we facilitate our introspection model to predict object-wise FNs, where the scales and locations of FN objects are characterized by bounding boxes. We believe object-wise FN predictions are more constructive for autonomous driving to use in decision-making.

### 2.2. Online Performance Prediction

In recent years, there has been an increasing amount of literature on online performance prediction for object detectors. Among them, some studies [4,11] aimed to predict relevant metric values of the detections on a test image. For example, Rahman and Niko [4] proposed a cascaded ordinal classifier to monitor the performance of an object detector by predicting the mean Average Precision (mAP) of a short sequence of image frames.

Some other studies attempted to quantify the qualities of the outputs of a given object detector. Gupta and Carlone [7] introduce an online monitor, called ATOM, to predict the losses in the outputs of a human-pose-and-shape reconstruction network. Schubert et al. [26] proposed a post-processing method to quantify the uncertainty and quality of detection results. They use handcrafted metrics to train a post-processing model, and predict an Intersection-over-Union (IOU) score for each detection, as well as a probability to distinguish between TPs and FPs.

In recent years, Dropout Variational Inference [27] has made Bayesian Neural Networks (BNN) a tractable solution to provide epistemic uncertainty quantifications for deep neural networks, and Miller et al. [8] applied this method to object detection for the first time. Currently, increasing attention [1,9,10,28] has been devoted to quantify uncertainties in the results of object detectors.

While the above methods can predict the performance metrics on an input image, or the qualities and uncertainties of each detected object, they cannot deal with FN objects, since FN objects are not among the detection results.

### 2.3. FN Prediction

For safety-critical applications such as autonomous driving, FN objects pose serious safety concerns and tend to cause fatal accidents. Nevertheless, the online FN object prediction problem has been rarely studied. In this subsection, we introduce three relative studies we found in the literature.

Rahman et al. [12] propose a method, called False Negative Detector (FND), to identify traffic signs that have been missed by a traffic sign detector. Based on the observation that some excited regions in the internal feature maps of the traffic sign detector correspond to FNs, they select several feature maps from the detector, and use them to train a classifier to predict whether there are FNs in regions without detection results. Limitations of this method lies in that the model structure and its internal outputs of the given detector can be unattainable in practical deployment, and that not all FNs will surely have activated regions in feature maps of a given detector.

Instead of analyzing the internal features of a given object detector, Ramanagopal et al. [2] think that inconsistencies in detections on two similar images indicate clues for FNs. Therefore, they propose a FN prediction method that uses temporal and stereo inconsistent detections as hypotheses of FNs, and choose 11 features to train a binary classifier to identify real FNs from these hypotheses. While the assumption in this method is intuitively consistent with our common observations, it relies on the detection facility of the given detector to raise FN proposals. This means objects missed in both of the similar images will have no chance to be predicted as FNs. Besides, additional tracking algorithm and stereo detections are needed to raise FN proposals, which increases the computation cost and algorithm complexity.

Similar to the methods mentioned above, Rabiee and Biswas [13] also train a classifier to determine the probability of an image patch to be FN, FP, TP or True Negative (TN). Instead of providing object-wise predictions, they aim to predict which image patches are likely to cause failures in a stereo vision-based obstacle avoidance system. They use a supervisory sensor in addition to the stereo vision sensor, and the unreliable patch proposals are those divergent in projected plans generated by these two types of sensors.

Overall, these studies provide valuable insights of the characteristics of FNs, as well as feasible ways to find clues for FNs. However, due to the empirical assumptions or handcrafted features of FNs they rely on, the upper bonds of their performance are limited, and some of these methods lack generalization facility to different object detectors. Consequently, none of the above methods can make object-wise FN predictions based on non-handcrafted FN features for black-box object detectors.

## 3. Problem Formulation

### 3.1. FN Object Definition

According to [29], a FN is the error where the test result incorrectly fails to indicate the presence of a condition when it is present. Based on this explanation, a FN object in object detection application can be described as an object with no matching detection result in the final outputs of an object detector.

In this work, we specifically define FN objects as ground truth labels that are FNs according to the hard evaluation mode of the widely adopted KITTI Benchmark [14]. In practice, we relax the overlap IOU threshold from 0.7 to 0.5 as other studies do [2,12].

### 3.2. FN Prediction Problem Formulation

Instead of using handcrafted features, we use a CNN-based introspective FN predictor to extract FN features and make object-wise predictions.

(1) FN Feature Extraction

Let $\Phi :\;R^{W\times H\times 3}\to R^{W/r\times H/r\times K}$ be the CNN model of the FN predictor, where r refers to its down-sampling ratio, K is the number of channels in its output, W and H are the width and height of an input image respectively. To better explain the extraction of FN features, we suppose that the CNN model has a backbone B and prediction branch(es) P as most of the CNN models do.

Taking an image $I\in R^{W\times H\times 3}$ as input, the generated feature maps of the ith layer $\ell_{i}$ in B are denoted as $\chi_{i}$, and can be defined in a convolutional way:

(1) $$\chi_{i}=\ell_{i}\left(\chi_{i-1}\right)=\ell_{i}\left(\ell_{i-1}\left(\ell_{i-2}\left(\ldots \ell_{2}\left(\ell_{1}\left(I\right)\right)\right)\right)\right),$$

where $i\in {Ν}_{l}=\left\{i\;|\;i\in Z^{+}\;with\;i\leq n\right\}$, n is the number of layers in B, and ${Ν}_{l}$ is the set of indexes of the layers in B.

Let $X_{pred}=\left\{\chi_{j}\;|\;j\in {Ν}_{l}\right\}$ be the set of feature maps responsible to extract FN predictions, the extracted FN features can be obtained as follows:

(2) $$Feature_{FN}=\Phi (I;\Theta )=P(X_{pred}),$$

where Θ is the parameter set including all the parameters in Φ.

(2) Object-wise Prediction

Let ψ be the object-wise decoder to get FN predictions from $Feature_{FN}$, the online FN prediction process is then expressed as follows:

(3) $$\hat{O_{FN}}=\psi \left(Feature_{FN}\right)=\psi \left(\Phi \left(I;\;\Theta \right)\right),$$

where $\hat{O_{FN}}$ is the set of decoded object-wise FN predictions:

(4) $$\hat{O_{FN}}=\left\{\;\hat{O_{FN}^{k}}\;|\;\hat{O_{FN}^{k}}=\left(\;\hat{C},\;\hat{bbox}\right),\;\;k\in Z^{+}\right\},$$

with

(5) $$\hat{bbox}=\left(\;\hat{u},\hat{v},\;\hat{h},\hat{w}\right),$$

$\hat{C}$ is the predicted category label distribution, $\hat{bbox}$ is the predicted bounding box for a FN object, with (u, v) being the center coordinates, and (h, w) being its height and width respectively.

Given a black-box object detector D, and a dataset $S=\left\{S_{1},\;\ldots ,\;S_{{Ν}_{d}}\right\}$ with ${Ν}_{d}$ samples, the FN objects missed by D can be denoted as $O_{FN}=\left\{O_{FN}^{1},\;\ldots ,\;O_{FN}^{{Ν}_{d}}\right\}$. Therefore, the training objective of the CNN-based introspective FN predictor is to find Θ for which $\hat{O_{FN}^{k}}\left(S_{k}\right)$ is close to $O_{FN}^{k}$ for all $k\leq {Ν}_{d}$, and ideally Θ is expected to suit any samples encountered in deployment. This is achieved by the proposed framework in our work, which will be introduced in detail in Section 4.

## 4. Method

### 4.1. Overall Framework

**Figure 4:**
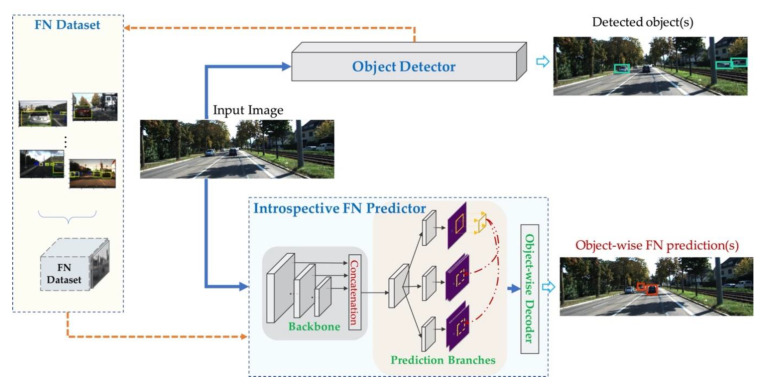
The introspective FN prediction framework proposed in this work. The yellow dotted lines refer to the generation of the FN dataset and its use in the training process, and the blue solid lines refer to the inference process of both object detections and online FN predictions.

The proposed introspective FN prediction framework is depicted in Figure 4, which mainly consists of three parts: the given black-box object detector, the FN dataset, and the introspective FN predictor.

The FN dataset is formed with image samples containing FN objects that are missed by the given black-box object detector. These FN objects are defined according to the definition in Section 3, and will further be used to train and evaluate the introspective FN predictor.

The given black-box object detector is used to collect image samples with FN objects to form the FN dataset, and to produce object detection results for autonomous driving during the inference process. It is worth noting that the proposed framework has no prerequisite for the black-box object detector, it can be any object detector applied in a given autonomous driving system.

The FN predictor consists of three parts: a CNN model with a backbone and three prediction branches to extract FN features, and an object-wise decoder for generating object-wise FN predictions. Details of the introspective FN predictor will be explained in Section 4.2 and Section 4.3.

### 4.2. Introspective FN Predictor

Instead of using handcrafted FN features, we design and train a CNN-based FN predictor in an introspective way, and use it to make object-wise predictions based on the FN features it learns. As depicted in Figure 4, the FN predictor is constructed with a backbone, prediction branches, and an object-wise decoder.

#### 4.2.1. Backbone

The backbone of the FN predictor is mainly composed of convolutional layers for image processing. In practice, we can use a pre-trained model structure, such as VGG16 [30], ResNet [31], and MobileNet [32].

As FN objects can be small and inconspicuous, in order to obtain more comprehensive information about FN objects, we can concatenate the feature maps of different resolutions at the end of the backbone, as depicted in Figure 4, and make these feature maps responsible to extract FN predictions. This is because the feature maps with higher resolutions are supposed to provide more precise location information, while the feature maps with lower resolutions are supposed to contain more complex semantic information [20].

We use the light-weight MobileNet [32] as the backbone when constructing the FN predictor for experimental validation in Section 5. Specifically, we denote the feature maps in the pre-trained MobileNet [32] model as $\phi_{2}$, $\phi_{3}$, $\phi_{4}$ and $\phi_{5}$ as [20] do, and let $X_{pred}=\left\{\phi_{3},\;\phi_{4}\;,\;\phi_{5}\right\}$ be the set of feature maps responsible to extract FN predictions. Although the construction of $X_{pred}$ can be optimized by experimentally comparing different combinations of feature maps in the backbone, the optimal combinations can be different for different backbone structures we choose to use in practice, and suboptimal selections will not affect the validation of the proposed framework. Therefore, we do not take finding the optimal combination for the MobileNet backbone as our focus in this paper.

#### 4.2.2. Prediction Branches

The design of prediction branches and their outputs depend on how we want to describe the predicted FN objects. As mentioned in Section 3, we want the prediction branches to provide the category label C, the center position (u, v), and the height h and width w of each FN object.

**Figure 5:**
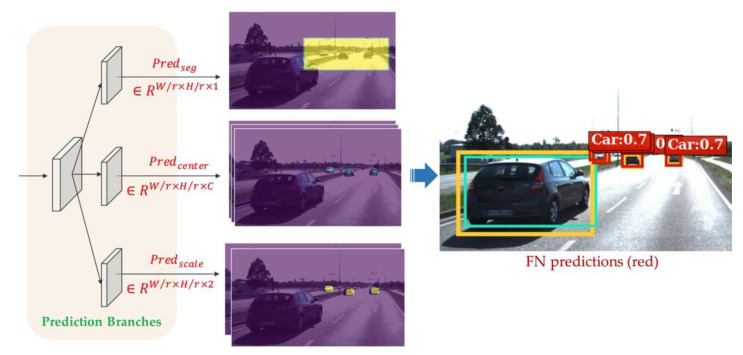
Outputs of prediction branches and the corresponding object-wise FN predictions. Object-wise FN predictions are obtained from the output feature maps, and are denoted as red solid rectangles with category labels in the right image. For better understanding of these FNs, the ground truth objects and detections from the black-box object detector are also provided, which refer to the yellow and green rectangles respectively. Please note that here we crop unrelated background of the image and the feature maps for better visualization.

Inspired by anchor-free object detectors [19,20], we locate FN objects by their center points in the image, since the center point of an object is considered to be a robust and comprehensive feature [19]. Besides, we describe the model outputs in the form of feature maps, so that each FN proposal can be represented by the output values at the same pixel coordinates of different feature maps, as depicted in Figure 5. In doing so, the model can conveniently express variable number of FN objects in different input images.

Figure 5 describes the prediction branches and their outputs, from up to down, the first branch is responsible for predicting the segmentation heatmap $Pred_{seg}$ that contains all the FN objects in its foreground. It will work with other branches in a cooperation mechanism, so that the distinct foreground-background imbalance problem of FN objects can be alleviated by narrowing down the searching spaces. This mechanism will be introduced in detail in Section 4.3. The second and third branches are defined in the same way as in [20], which are for predicting the center point heatmap $Pred_{center}$ and scale map $Pred_{scale}$ respectively.

#### 4.2.3. Object-Wise Decoder

To get the inference results of FN bounding boxes, in the post-processing step, we use the object-wise decoder to extract object-wise FN predictions. The decoder treats the center score predictions in $Pred_{center}$ to be the classification results, and filter the center heatmap $Pred_{center}$ with a threshold of 0.5. For the remained pixels, the decoder constitutes bounding boxes based on the heights and widths in the predicted scale map $Pred_{scale}$, as depicted in Figure 5. Finally, highly overlapped bounding boxes will be removed by Non-Maximum Suppression (NMS), and the remaining ones will be exported as the results of FN prediction.

### 4.3. FN Predictor Loss Function

To alleviate the foreground-background imbalance of FN objects, and to promote the training process of the FN predictor, we design the loss functions in a way that these branches can be associated and cooperated.

#### 4.3.1. Branch Cooperation Mechanism

As mentioned earlier, FN objects usually suffer from severe foreground-background imbalance problem, which makes it difficult for the proposed FN predictor to extract effective features from the whole image.

Chen et al. [33] analyze the existing methods on solving the foreground-background imbalance problem, and hold that reducing the searching space of an object detector may solve this problem. Inspired by this idea, we add an auxiliary segmentation task to the proposed FN predictor, the output of which is a segmentation map that ideally contains all the FN objects in its foreground. In this way, the Region of Interest (ROI) is narrowed down from the entire image to the foreground of the segmentation map, and the foreground-background imbalance problem can be greatly alleviated if other branches only focus on the ROIs in the training process.

To achieve this mechanism, we treat the segmentation output as a contribution mask, and apply it to the losses of the rest branches, so that different branches can be coupled with each other, and encouraged to learn consistent features during the training process. The design of losses for each branch is described below. Please note that the ground truth training labels we use share the same format with the output feature maps described in Figure 5.

#### 4.3.2. Segmentation Loss

For the segmentation branch, we use dice loss as the loss function, and express it by Equation (6):

(6) $$Loss_{seg}=1-Dice\left(GT_{seg},\;Pred_{seg}\right),$$

where $GT_{seg}\in R^{W/r\times H/r\times 1}$ is the ground truth segmentation map, and Dice(.) is the dice coefficient function. The foreground of $GT_{seg}$ is the smallest envelope rectangle containing all the FN objects in the image. In practice, we expand the foreground region by a ratio of ρ, in case the FN predictor needs to infer from the background. ρ is experimentally set as 0.2.

#### 4.3.3. Center Loss

For the center point branch, we adopt the focal cross entropy loss in [20], and formulate the center loss as:

(7) $$Loss_{center}=-\frac{1}{K_{s}}\sum_{i=1}^{W/r}\sum_{j=1}^{H/r}w_{ij}CE\left(p_{ij},\;{\hat{p}}_{ij}\right)$$

where $K_{s}$ is the number of pixels in the foreground of the rounded $Pred_{seg}$, CE(.) is the cross entropy function, $w_{ij}$ is the focal weight at pixel (i,j), which is defined slightly different from [20]:

(8) $$w_{ij}=\{\begin{array}{c} exp\left(\alpha \left(p_{ij}-{\hat{p}}_{ij}\right)\right),\;\;p_{ij}>0\\ \left(1-p_{ij}\right)\;exp\left(\beta {\hat{p}}_{ij}\right),\;\;p_{ij}\leq 0 \end{array}$$

where $p_{ij}\in GT_{center}$, and ${\hat{p}}_{ij}\in Pred_{center}$, $GT_{center}\in R^{W/r\times H/r\times 1}$ is the ground truth center heatmap assigned by Gaussian kernels following [34], α and β are hyper-parameters, which are experimentally set as 4 and 2 respectively.

#### 4.3.4. Scale Loss

We hope the training objectives can be more consistent with the evaluation metrics of FN predictions, therefore, instead of using popular regression losses, such as L1 loss, and L2 loss, we use the GIOU [35] to measure the overlaps between predicted bounding boxes and ground truth ones, in this way, different branches can be better associated with each other in the training process. The scale loss is formulated as Equation (9):

(9) $$Loss_{scale}=\frac{1}{K_{p}}\sum_{i=1}^{W/r}\sum_{j=1}^{H/r}\left(1-GIOU\left(B_{ij},\;{\hat{B}}_{ij}\right)\right)$$

where $K_{p}$ is the number of positive pixels in the rounded $GT_{center}$. $B_{ij}$ is the ground truth bounding box at pixel (i,j), and ${\hat{B}}_{ij}$ is the predicted bounding box at pixel (i,j), which is formed by predicted height ${\hat{h}}_{ij}$ and width ${\hat{w}}_{ij}$ in the feature map $Pred_{scale}$.

#### 4.3.5. Total Loss

The total loss is the weighted sum of the above loss functions, as expressed by Equation (10):

(10) $$Loss_{total}=\lambda_{seg}Loss_{seg}+\lambda_{center}Loss_{center}+\lambda_{scale}Loss_{scale},$$

where $\lambda_{seg}$, $\lambda_{center}$, and $\lambda_{scale}$ are experimentally set as 2.5, 6 and 0.5 respectively.

### 4.4. Implementation

In this subsection, we explain the implementation details of our proposed framework.

During the preprocessing stage, we adopt the data augmentation pipeline in [15] for the training process. Taking the hardware conditions into consideration, we set the image resolution of the model input to be 720 · 300. After the data preprocessing, ground truth FN objects with height or width less than a threshold will be ignored in the training process. In practice, we set this threshold to be 1 pixel.

During the training stage, we train the FN predictor with a batch size of 16 on a Geforce 2080ti GPU for 300 epochs. We use the Adam optimizer, and set the initial learning rate to be 1e-4, and decrease it by 10% per 10 epochs. The platform we use is Keras/Tensorflow 1.9, CUDA 9.0, and cuDNN v7.0.5.

## 5. Experiments

In this section, we conduct extensive experiments and analysis to evaluate the performance of the proposed introspection framework. While the proposed framework can perform multi-class FN predictions, we apply it as a FN car predictor for autonomous driving, and present all the analysis for the Car category.

### 5.1. Experimental Settings

#### 5.1.1. Object Detector

We apply the proposed framework to predict FNs of the popular SSD [15] detector, the weight model we use is the original SSD300 provided in [15]. The specific model structure is only used to realize the object detection function in the proposed framework, and is not used in the FN predictor, so for the FN prediction task, the SSD object detector is indeed a black box.

#### 5.1.2. Dataset

The application scenes in autonomous driving are often different from the training datasets of object detectors, which is a main source of unreliable detections. Taking this problem into consideration, we use the challenging KITTI 2D Object dataset to construct the KITTI 2D FN dataset, since the SSD detector we use is trained on PASCAL VOC [36]. By evaluating SSD on KITTI 2D Object dataset, we obtained the FN dataset, denoted as 2D-FN, which contains 5184 samples with 13,265 FN car labels, we randomly split this dataset with 0.7:0.15:0.15 ratio for training, validation and test.

To further verify the effectiveness of our FN predictor on a larger dataset than the 2D-FN test dataset, we evaluate the FN prediction performance of the trained FN predictor on the KITTI tracking dataset as well. We denote the KITTI tracking dataset as TRK, which is short for tracking, and in order to construct the corresponding TRK-FN dataset, we evaluate the detection performance of the SSD detector on the TRK dataset, finding 12078 FNs in total of 5060 samples. Please note that TRK-FN is only used for testing.

**Figure 6:**
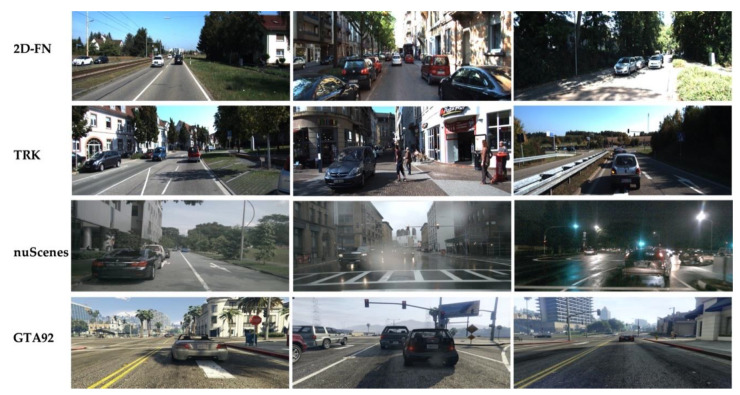
Samples from the test datasets we adopted. While the 2D-FN and the TRK share similar weather conditions and driving scenes, the nuScenes and the GTA92 dataset both show distinct domain disparities caused by different driving conditions and driving scenes.

We also conduct tests on the challenging nuScenes dataset [37] and the GTA92 dataset [2] to further prove the effectiveness, and make comparisons with previous literature. The nuScenes dataset is a recent urban driving dataset collected in the real world with approximately 1.4M camera images and 1.4M object bounding boxes, we use the images from its front camera and take all the 25655 samples with car labels into consideration. The GTA dataset [2] is a tracking dataset generated from a game engine, which contains 104 sequences. We use the GTA92 dataset [2], which contains 92 sequences in the GTA dataset, and 66056 samples with car labels. Although these two datasets are also intended for autonomous driving, compared to the 2D-FN and the TRK which are from the KITTI Benchmark, there exist distinct domain disparities caused by different driving scenes and conditions, as depicted in Figure 6. This makes the input distributions of these two datasets shifted from the training distribution of the 2D-FN dataset, which is often the case when we deploy object detectors in the real world.

In contrast to the existing FN prediction methods [2,12,13], we do not need to retrain the given object detector on the chosen dataset, since the performance of the black-box object detector will not affect the predictions for FN objects.

### 5.2. Evaluation Metrics

The proposed framework is evaluated from the following three aspects.

#### 5.2.1. Bounding Box Level FN Prediction Accuracy

Since the proposed framework can provide object-wise FN predictions $\hat{O_{FN}}$, we adopt the widely used evaluation metrics in the field of object detection, and change the evaluation targets from car labels to all the FN car labels missed by the given object detector. Specifically, we use Precision, Recall, Average Precision (AP), and F1 score, and define them for FN evaluation as follows:

For FN Precision,

(11) $$Precsian_{FN}=\frac{TP_{FN}}{TP_{FN}+FP_{FN}}$$

where $TP_{FN}$ refers to the number of true-positive FN objects in the prediction results, and $FP_{FN}$ refers to the number of false-positive FN objects in the prediction results.

For FN Recall,

(12) $$Recall_{FN}=\frac{TP_{FN}}{TP_{FN}+FN_{FN}}$$

where $FN_{FN}$ refers to the number of FN objects which are expected to be predicted, but are not in the FN prediction results.

For FN AP,

(13) $$AP_{FN}|R_{FN}=\frac{1}{\left|R_{FN}\right|}\;\sum_{r_{FN}\in R_{FN}}\rho_{FN\_interp}\left(r_{FN}\right),$$

with

(14) $$\rho_{FN\_interp}\left(r_{FN}\right)=\underset{r_{FN}^{\prime }:r_{FN}^{\prime }\geq r_{FN}}{\max}\rho_{FN}\left(r_{FN}^{\prime }\right)$$

where $\rho_{FN}\left(r\right)$ is the FN precision at FN recall $r_{FN}$, and $R_{FN}$ is the set of sampled FN Recalls. In practice, we set $R_{FN}=\left\{\frac{1}{40},\;\frac{2}{40}\;,\frac{3}{40}\;,\ldots ,\;1\right\}$ as the KITTI Benchmark [14] do.

For FN F1 score,

(15) $$F1_{FN}=\frac{2*Precision_{FN}*Recall_{FN}}{Precision_{FN}+Recall_{FN}}$$

Besides, we take the number of true-positive FN predictions $TP_{FN}$ and the number of false-negative FN predictions $FN_{FN}$ as metrics of bounding box level FN prediction performance.

#### 5.2.2. Pixel Level FN Prediction Accuracy

The bounding box level evaluations can be very different when using different overlap IOU thresholds to define $TP_{FN}$, thus we add the pixel-level evaluation of FN predictions, where the evaluation targets are FN pixels inside the bounding boxes.

In other words, the set of true-positive FN pixels in an image is defined as:

(16) $$TP_{FN}=\left\{pixel_{i}|\;pixel_{i}\in \left(Gt_{FN}\cap Pred_{FN}\right)\right\}$$

where $Gt_{FN}$ is the set of ground truth FN objects in this image, and $Pred_{FN}$ is the set of predicted FN objects in the same image. The set of false-positive FN pixels in the image is then defined as:

(17) $$FP_{FN}=\left\{pixel_{i}|\;pixel_{i}\in Pred_{FN},\;and\;\;pixel_{i}\notin Gt_{FN}\right\}$$

and

(18) $$FN_{FN}=\left\{pixel_{i}|\;pixel_{i}\in Gt_{FN},\;and\;\;pixel_{i}\notin Pred_{FN}\right\}$$

In this way, the pixel level FN precision and recall can be calculated according to Equations (11) and (12).

To further analyze the FN feature identification ability of the proposed framework, we calculated the semantic ratio of predicted FN pixels to the whole image pixels, and compared it with the ratio of ground truth FN pixels.

#### 5.2.3. Quantitative Improvements to Object Detection

As is done in [2] and [12], We analyze the effectiveness of FN prediction by quantifying the object detection improvements of the given object detector when taking the FN predictions into consideration.

Please note that for all the bounding box level metrics including the quantitative improvements to object detection task, statistical results are calculated using the hard evaluation mode of the widely adopted KITTI benchmark [14]. In practice, we set the overlap IOU threshold to be 0.5 in order to be consistent with the definition of FN objects.

### 5.3. Quantitative Evaluation Results

In contrast to our work, previous FN prediction methods usually contain two stages: finding the FN proposals, and then training a classifier to classify the true FNs from these proposals. Thus, in their evaluation experiments, they mainly use the classification accuracies of the FN proposals to describe the effectiveness of their methods.

On the contrary, we evaluate our method on all the ground truth FN labels in the test dataset, instead of FN proposals, as defined in Equation (12). This makes the FN prediction accuracy metrics used in our work not equivalent to the classification accuracies used in the literature. Due to the fact that our method does not need to make FN proposals, or to use a classifier, we are not able to provide a classification accuracy for FN proposals as previous methods do. On the other hand, due to limited open-source data we can get, it is infeasible for us to calculate the evaluation results of the previous methods based on our metrics.

Thus, comparison of the metrics of bounding box level and pixel level FN prediction accuracies to other approaches is infeasible in this subsection. Instead, we conduct extensive experiments and analysis on different aspects of the proposed method, and compare the FN prediction performance on different datasets.

Nevertheless, comparisons of the quantitative improvements to object detection tasks are available. Ref. [2] provides their evaluation results of this metric on a test dataset that is very different from their training dataset. Thus, using the same evaluation settings in [2], we also test the facility of our method on the nuScenes and the GTA92 dataset, which have distinct domain disparities compared to our training dataset.

The experiment data on KITTI Benchmark is accessible in the Supplementary Materials, and the full experiment data can be downloaded from the link provided in the Supplementary Materials. The experimental results are explained below.

#### 5.3.1. Bounding Box Level FN Prediction Accuracy

The FN bounding box prediction accuracies are shown in Table 1, our framework achieves 81.95% precision for 88.10% recall with 0.5 IOU threshold, and correctly predicts 1703 FN objects which are missed by the given object detector.

Depending on the usage of FN predictions, the spatial accuracy of the bounding boxes can be relaxed if they are only used for alarms, so we also provide evaluation results under different IOU thresholds in Table 1.

#### 5.3.2. Pixel Level FN Prediction Accuracy

We also calculate the pixel level FN prediction accuracies, since they can demonstrate the FN prediction facility in a more direct way. The evaluation results show that the proposed method achieves 71.71% Precision for 83.47% Recall on the pixel level, and among all the test images, the average semantic ratio of predicted FN pixels to all pixels in the image is 2.47%, which is very close to the ground truth semantic ratio of 1.96%.

**Figure 7:**
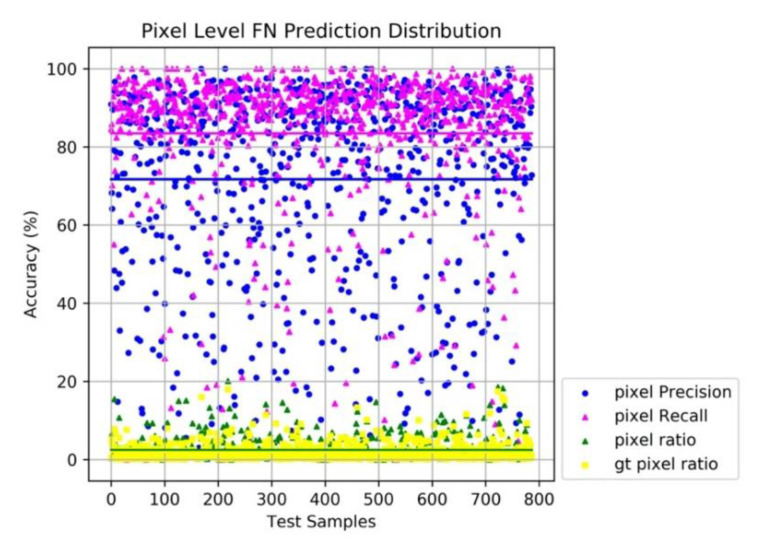
Pixel level FN prediction performance distribution on the test dataset of 2D-FN. Each point in the figure stands for a metric value on a test image, and the solid lines refer to the average values among all the test samples.

The distribution of FN pixel prediction accuracies in each test images are shown in Figure 7.

#### 5.3.3. Quantitative Improvements to Object Detection

We gather the results of the predicted FN bounding boxes and the object detections of the given black-box, and apply NMS on them to take the FN predictions into consideration. Based on the domain disparities, we divide the test datasets into two groups. Group 1 refers to the 2D-FN test dataset and the TRK-FN, which, like our training dataset, are both from the KITTI Benchmark. Group 2 refers to the nuScenes and the GTA92, where test samples are very different from our training dataset.

(1) Performance on the test datasets in Group 1

**Figure 8:**
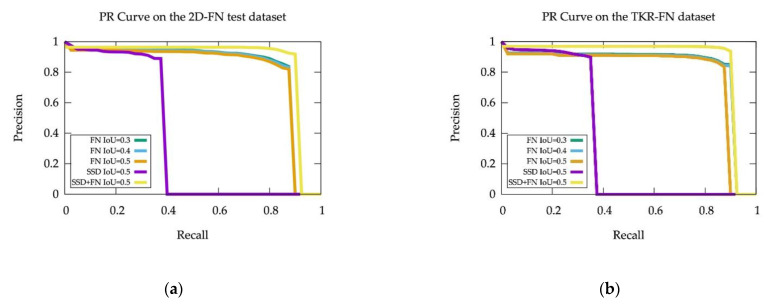
PR Curves on different datasets. (**a**) PR Curves on the 2D-FN test dataset. (**b**) PR Curves on the TRK-FN dataset. PR Curves of the FN prediction performance are labeled as FN, the object detection performance of the given object detector are labeled as SSD, and the object detection performance when taking FN predictions into consideration are labeled as SSD + FN. Therefore, the quantitative improvements to the object detection performance can be visualized by comparing the SSD + FN PR Curve with the SSD curve.

The Precision-Recall (PR) Curves on the 2D-FN test dataset and the TRK-FN dataset are shown in Figure 8.

The detailed evaluation results are listed in Table 2, the results show that our framework improves the detection performance by more than 50% in AP, and correctly predicts 1664 and 10697 FN objects missed by the given object detector in the 2D-FN test dataset and the TRK-FN dataset respectively.

(2) Performance on the test datasets in Group 2

The nuScenes and the synthetic GTA92 both have massive test samples and their driving scenes are very different from our training dataset. We adopt the F1 score as [2] do, and provide the evaluation results of our FN predictor as well as the comparison with Refence [2] in Table 3.

#### 5.3.4. Different Input Resolutions

We find that the precision and recall of the FN predictions will be decreased by nearly 10% if we decrease the training resolution from 720 x 300 to 300 × 300. We also try 1200 × 720 resolution, with the corresponding batch size decreasing from 16 to 4 due to hardware limitation, the training process is barely improved under this circumstance.

### 5.4. Qualitative Results

**Figure 9:**
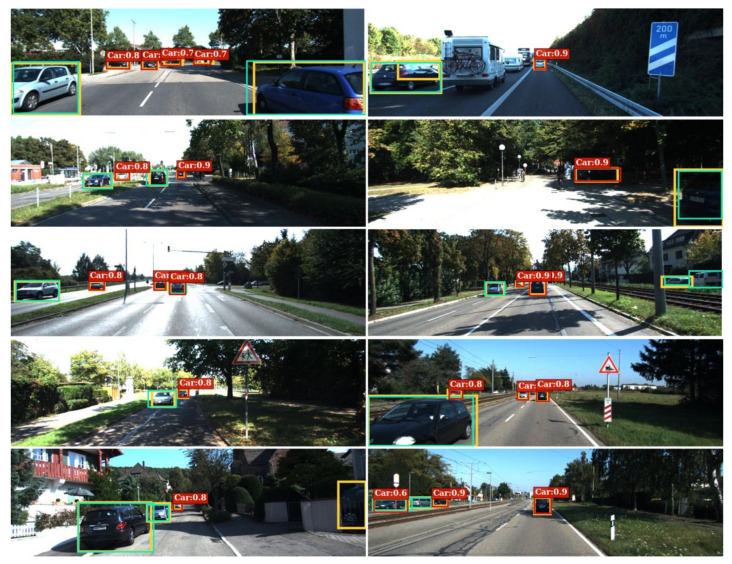
Qualitative examples from the test dataset. The FN predictions made by the proposed framework are denoted as red bounding boxes with car labels and the corresponding confidences. It can be seen that among all the ground truth car labels (yellow), the given object detector makes good detections (cyan) on the distinctive ones, while the proposed framework manages to predict the FN objects (red) missed by the detector even under perceptually degraded conditions.

We visualize qualitative examples in diverse scenes in Figure 9. It is worth noting that all these images are not included in the training phase. The results demonstrate the FN prediction effectiveness of the proposed introspective FN predictor.

### 5.5. Discussion

The quantitative evaluation results in Section 5.3 and the qualitative results in Section 5.4 verify the FN prediction facility of our FN predictor, thus proves the effectiveness of the FN feature extraction by the proposed introspective framework.

**Bounding box level FN prediction accuracy**: The FN predictor achieves more than 88% recall on predicting FN objects in the 2D-FN test dataset and the TRK-FN dataset, and the prediction accuracies are higher with lower IOU thresholds when we use the FN predictions for alarms in autonomous driving. This indicates our FN predictor retrieves most of the FN objects missed by the given object detector, and can help to avoid traffic accidents by making autonomous driving system take the FN predictions into consideration during its deployment.

**Pixel level FN prediction accuracy**: The average semantic ratio of predicted FN pixels to all pixels in the image is very close to the ground truth semantic ratio of 1.96%. This suggests that although the ground truth FN pixels only account for a very small proportion of the image, the proposed method does not suffer from severe foreground-background imbalance owing to the multi-branch cooperation mechanism. Besides, instead of confusing the FN pixels with pixels inside the detected cars by SSD, or the pixels of background, our proposed method manage to predict them correctly. This suggests that the introspective FN predictor is able to learn the detection characteristics of the given black-box object detector, and extract effective FN features.

**Quantitative Improvements to object detection**: From Table 2 we can see that while object detection precisions are barely improved, the recalls, APs, and F1 scores have made significant improvements, which means the proposed FN prediction framework can reduce FNs and improve the safety of autonomous driving.

We noticed that the precisions of the given object detectors are barely improved after taking the FN predictions into consideration in Table 2. According to Equation (11), the direct reason is FP predictions for FNs are added to the object detection results. This can be the result that, in the object-wise decoder, the filtering method of the center heatmap is not distinguishable enough, and the original NMS we use might impact the prediction precision [38], especially with dense targets [39]. Therefore, more effective post-processing methods should be considered in future work.

Table 3 further proves the effectiveness of the proposed FN predictor on both the real world and the synthetic datasets. Our test datasets are larger and more diverse than the TRK dataset used in [2], and our proposed FN predictor improves the F1 score of detection performance by 4.84% on the GTA92 dataset, and by 8.22% on the nuScenes dataset, which are higher than the improvements achieved by [2]. This indicates that the FN features learned by our proposed framework are representative, and can well reflect the detection characteristics of the given object detector.

**Different input resolutions**: Based on our experiments on different input resolutions, higher resolution with large batch size is supposed to get better FN prediction performance, this might be because it allows a training batch to contain more detailed and comprehensive FN features, which facilitates the learning process of the FN predictor.

## 6. Conclusions

In this paper, we present a novel introspective framework to perform online FN prediction for black-box object detectors. Unlike previous methods, which depend on empirical assumptions or handcrafted FN features to identify FNs, our approach provides a new perspective where the online FN prediction problem can be formulated as an introspection model. Thus, instead of “defining” FN features by ourselves, or making assumptions on what FN objects should share in common, we let the FN predictor to “learn” to identify FN features for a given object detector based on the introspective framework we propose. This makes our method not subject to the limitations caused by handcrafted features or empirical assumptions for FNs, and not need to raise FN proposals by using additional algorithms as most previous methods have to. To achieve that, we extend the original concept of introspection to object-wise FN prediction, and propose a multi-branch cooperation mechanism to address the distinct foreground-background imbalance problem in FN prediction.

Collectively, our proposed framework is the first to provide object-wise FN predictions that are not based on handcrafted features or empirical assumptions for FNs, besides, our framework is able to be applied to black-box object detectors. Extensive experimental results verify its effectiveness in helping improve the object detection task, and furthermore the safety of autonomous driving. We plan to improve the introspection ability of our framework by taking the uncertainties of the FN predictions into consideration, and incorporate the uncertainties with the object-wise decoder to further improve the FN prediction accuracy in our subsequent research work.

## Figures and Tables

**Table 1:** Bounding box level FN prediction accuracy.

FN Dataset	IOU Threshold	Precision	Recall	AP	F1 Score	TP	FN	FP
2D-FN test	0.5	81.95%	88.10%	80.96%	84.91%	1703	230	375
	0.4	83.16%	89.23%	81.64%	86.09%	1724	208	349
	0.3	83.71%	89.74%	81.94%	86.62%	1732	198	337
TRK-FN	0.5	83.49%	90.97%	79.69%	87.07%	10,601	1052	2096
	0.4	84.31%	91.49%	82.06%	87.75%	10,659	991	1984
	0.3	84.91%	91.77%	82.47%	88.21%	10,689	958	1900

**Table 2:** Quantitative improvements to object detection.

FN Dataset	Model	Precision	Recall	AP	F1 Score	TP	FN	FP
2D-FN test	SSD	88.93%	35.19%	36.49%	50.43%	1076	1982	134
	SSD + FN predictor	91.91%	89.43%	86.63%	90.65%	3691	318	237
	Improvement	**+2.98**%	**+54.24**%	**+51.44%**	**+40.22%**	**+2615**	**−1664**	**+103**
TRK-FN	SSD	89.85%	34.60%	34.34%	49.96%	6389	12,078	722
	SSD + FN predictor	93.71%	92.34%	87.31%	93.02%	16,659	1381	1118
	Improvement	**+3.86%**	**+57.74%**	**+52.97%**	**+43.06%**	**+10,270**	**−** **10,697**	**+396**

**Table 3:** Comparison of improvements to object detection on divergent distributional datasets.

Object Detector	SSD [2]	RCNN [2]	RRC [2]	SSD	SSD
Test dataset	TRK	TRK	TRK	GTA92	nuScenes
Improvement	+4.22%	+0.87%	+3.57%	**+4.84%** (Ours)	**+8.22%** (Ours)

## Data Availability

The data presented in this study are available in the Supplementary Materials.

## Supplementary Materials

The following are available online at https://www.mdpi.com/article/10.3390/s21082819/s1, Archive S1: Experiment_data.zip.
